# Protective Effects of Liquiritigenin against Citrinin-Triggered, Oxidative-Stress-Mediated Apoptosis and Disruption of Embryonic Development in Mouse Blastocysts

**DOI:** 10.3390/ijms18122538

**Published:** 2017-11-27

**Authors:** Chien-Hsun Huang, Wen-Hsiung Chan

**Affiliations:** 1 Department of Obstetrics and Gynecology, Taoyuan General Hospital, Ministry of Health & Welfare, Taoyuan City 33004, Taiwan; lithsunh@gmail.com; 2 Department of Bioscience Technology and Center for Nanotechnology, Chung Yuan Christian University, Chung Li District, Taoyuan City 32023, Taiwan; 3 Department of Medical Research, China Medical University Hospital, China Medical University, Taichung 40402, Taiwan

**Keywords:** liquiritigenin, citrinin, oxidative stress, apoptosis, embryonic development

## Abstract

The mycotoxin citrinin (CTN), a natural contaminant in foodstuffs and animal feeds, exerts cytotoxic and genotoxic effects on various mammalian cells and embryos. A previous investigation by our group revealed potentially hazardous effects of CTN on mouse oocyte maturation and pre- and post-implantation embryo development via the induction of apoptosis. The present study showed that CTN induces apoptosis and inhibits cell proliferation in the inner cell mass of mouse blastocysts. Notably, we observed for the first time that both these effects are suppressed by liquiritigenin (LQ). LQ is a type of flavonoid isolated from *Glycyrrhiza radix* with several biochemical and pharmacological activities, including antioxidant and anti-inflammatory properties. The preincubation of blastocysts with LQ clearly prevented CTN-induced disruption of pre- and post-implantation embryonic development and fetal weight loss, both in vitro and in vivo. CTN-induced damage processes directly promoted reactive oxygen species (ROS) generation, loss of mitochondrial membrane potential (MMP) and activation of caspase-9 and caspase-3, which were effectively blocked by LQ. Moreover, in an animal model, intravenous injection of dams with CTN (3 mg/kg/day) triggered apoptosis of blastocysts, disruption of embryonic development from the zygote to the blastocyst stage and a decrease in fetal weight. Pre-injection with LQ (5 mg/kg/day) effectively reduced apoptosis and impaired the cytotoxic effects of CTN on development. Our in vivo findings further confirm that CTN exposure via injection has the potential to impair pre- and post-implantation development, leading to apoptosis and the suppression of sequent embryonic development, which can be effectively prevented by LQ.

## 1. Introduction

Citrinin (CTN), a type of mycotoxin, is a secondary metabolite produced by several fungal species, such as *Penicillium* and *Monascus*. CTN contamination has been reported in several types of food, including corn, wheat, rice, barley and nuts, highlighting its harmful potential effects on human health [1,2]. *Monascus* fermentation products used in food colorants and flavor enhancers in the Orient, in addition to those used as health food or dietary supplements for the prevention of heart disease and the reduction of plasma triglyceride and cholesterol levels [3], have been shown to be contaminated with CTN [4,5]. Previous research has disclosed contamination levels of 0.28 to 6.29 μg/g CTN in lipid extracts from commercialized *Monascus* products [4]. The IC_50_ value of human HEK 293 cells treated with lipid extracts of *Monascus* products for 72 h is reported as 60 μM CTN [4]. Our previous studies demonstrated that CTN (30 μM) triggers mouse embryo apoptosis and developmental injury of mouse blastocysts, both in vitro and in vivo [6,7]. Moreover, CTN (5 μM) exerts significant inhibitory effects on mouse oocyte maturation, in vitro fertilization and early embryonic developmental injury in vitro and in vivo [8]. In view of these harmful injury effects of CTN, the development of effective strategies to prevent reproductive or embryonic toxicity remains an urgent unmet medical need.

Flavonoids, a main class of natural polyphenolic compound, are commonly found in vegetables, nuts, fruits, medicinal herbs and beverages [9]. Liquiritigenin (LQ; 7,4′-dihydroxyflavone), one of the flavonoid compounds isolated from *Glycyrrhiza radix*, has several biochemical and pharmacological properties, including anti-hyperlipidemic, anti-inflammatory, anti-allergic, and estrogenic activities [10,11,12,13]. Additionally, several studies have shown that LQ exerts anti-tumor activities via the induction of cell death in mammalian cell lines [14,15,16]. LQ is a major metabolite product of liquiritin that can be easily absorbed by the body [17]. Experiments in rats found that, after absorption, LQ is metabolized or breaks down into six metabolites localized to liver microsomes, including 7,3′,4′-trihydroxyflavone, a hydroxyl quinine metabolite, and two A-ring dihydroxymetabolites, 7,4′-dihydroxyflavone, and 7-hydroxychromone [18]. Previous studies have documented several protective effects of LQ against glutamate-induced apoptosis in hippocampal neuronal cells [19], heavy metal-induced toxicity in cultured hepatocytes [20], liver toxicity in rats [13] and methylglyoxal-induced cytotoxicity in osteoblastic MC3T3-E1 cells [21]. Additionally, LQ exerts cytoprotective effects against ROS-induced cell injury via its anti-oxidative activity [22]. The anti-oxidative activity of LQ derives from the phenolic hydroxyl group, which undergoes an abstraction reaction with hydrogen. The hydrogen atom is transferred and combines with reactive oxygen species for terminating the ROS-mediated chain reaction and consequently, free radical production [23]. Thus, LQ has been characterized as a potent ROS scavenger that can effectively prevent ROS-mediated cell injury and damage.

The cytotoxicity of CTN may be attributed to its function as an apoptosis inducer [6,7,24]. Numerous physical factors and chemical treatments can trigger apoptosis through augmenting intracellular ROS generation [25,26,27], and validating oxidative stress as an important upstream activator of cellular apoptotic signaling cascades. Importantly, CTN-induced ROS-mediated apoptotic processes in human hepatoma G2 cells could be attenuated by resveratrol [28]. These findings highlight natural chemical compounds with potent anti-oxidative capacity as potential candidates for development as health food products to prevent CTN-triggered health injury.

To ascertain whether LQ, a well-known anti-oxidative compound, is effective in protecting against CTN-triggered embryonic toxicity, its effects on apoptosis, proliferation, and blastocyst development of mouse embryos treated with CTN were examined. Pretreatment with LQ effectively blocked CTN-induced deleterious effects, including apoptosis, inhibition of cell proliferation, and retardation of pre- and post-implantation embryonic development, confirming its protective functions, both in vitro and in vivo.

## 2. Results

### 2.1. Protective Effects of LQ against CTN-Induced Injury to Blastocysts

**Figure 1:**
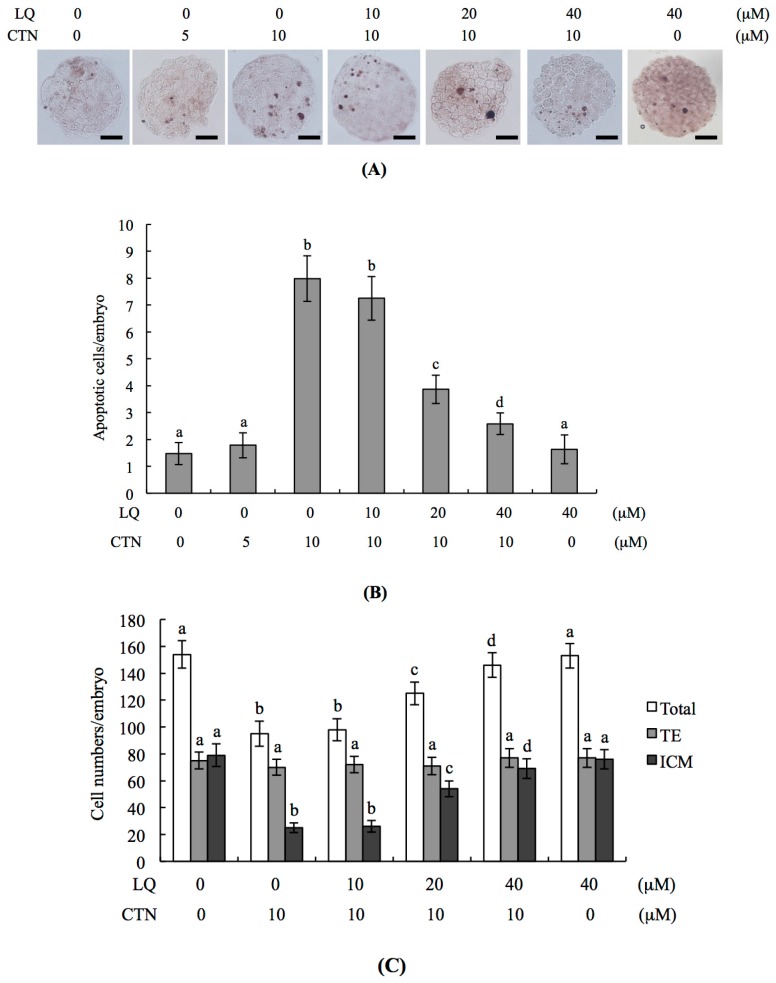
Effects of liquiritigenin (LQ) on citrinin (CTN)-induced apoptosis in blastocysts. Mouse blastocysts were pretreated with LQ at the indicated concentrations for 1 h, followed by treatment with or without CTN (5–10 μM) at the indicated concentrations for 24 h. Medium containing 0.5% dimethyl sulfoxide (DMSO) was used as the control (vehicle group). (**A**) Cell apoptosis in blastocysts was analyzed via terminal deoxynucleotidyl transferase dUTP nick end labeling (TUNEL) staining. TUNEL-positive cells (black) were visualized under a light microscope; (**B**) The mean number of apoptotic (TUNEL-positive) cells per blastocyst was counted; (**C**) Cell numbers of blastocysts were analyzed via differential staining and the number of inner cell mass (ICM) and trophectoderm (TE) cells per blastocyst calculated. Data were obtained from at least 240 blastocysts per group. Values are presented as means ± SD (standard deviation) of five determinations. Different symbols indicate significant differences at *p* < 0.05. The scale bar is 20 μm.

To determine whether LQ influences CTN-triggered apoptosis, blastocysts were pre-incubated with LQ (0–40 μM) for 1 h followed by CTN (0–10 μM) treatment for 24 h, and the level of apoptosis analyzed. Terminal deoxynucleotidyl transferase dUTP nick end labeling (TUNEL) data revealed significant apoptosis in the 10 μM CTN treatment group (Figure 1A). Quantitative analysis further showed that apoptosis is ~six-fold more prevalent in CTN-treated blastocysts than untreated controls (Figure 1B). The effects of LQ on CTN-triggered cell death were further investigated in mouse blastocysts. CTN-induced apoptosis was significantly attenuated upon pretreatment with 20–40 μM LQ (Figure 1A,B). We explored the protective effects of LQ on cells treated with CTN (10 μM) for 24 h by determining cell proliferation with the aid of differential staining for counting blastocyst cell numbers. Inner cell mass (ICM) cell numbers were significantly decreased in blastocysts treated with 10 μM CTN, compared to that in controls, while no marked differences were evident in the TE component (Figure 1C). These results indicate that CTN induces significant apoptosis in the ICM, but not TE, of mouse blastocysts. The CTN-induced decrease in the ICM cell number in blastocysts was effectively attenuated upon preincubation with 20–40 μM LQ (Figure 1C). Our results also showed that apoptosis and proliferation in the 40 μM LQ treatment group are not significantly different from those in the untreated control group, clearly indicative of no injurious effects of LQ on mouse blastocysts (Figure 1A–C). Based on these findings, we propose that CTN triggers apoptosis and reduction of cell proliferation in mouse blastocysts in vitro, both of which are effectively blocked by LQ.

### 2.2. CTN-Triggered Disruption of Blastocyst Development Is Prevented by LQ In Vitro

**Figure 2:**
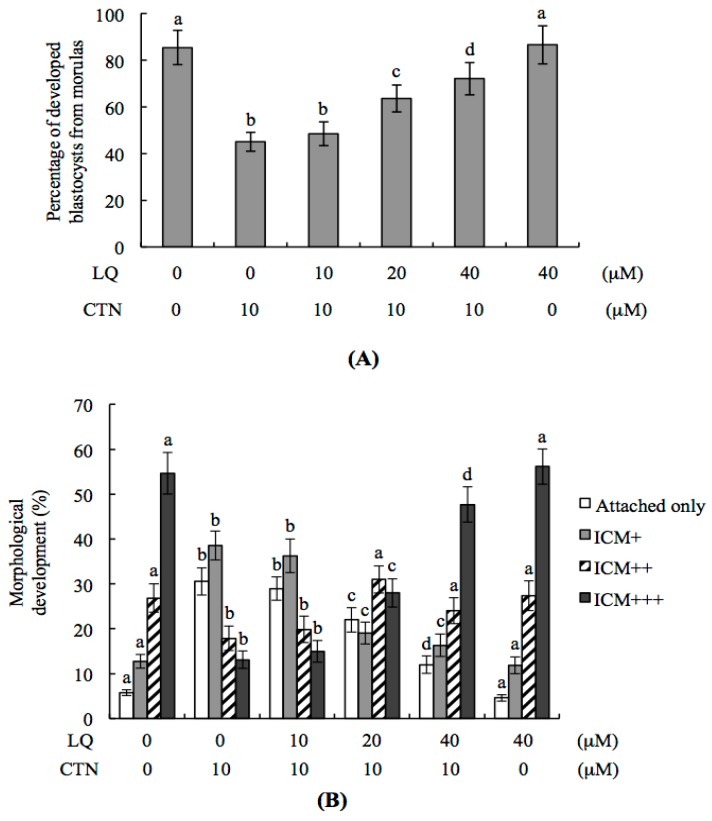
Effect of LQ on in vitro embryonic development of CTN-treated blastocysts. (**A**) Mouse morulae were preincubated with LQ (10–40 μM). After 1 h, embryos were further treated with or without 10 μM CTN for 24 h. Morulae were cultured for another 24 h at 37 °C, blastocyst numbers counted and the percentages calculated; (**B**) Mouse blastocysts were pre-incubated with LQ (10–20 μM) or vehicle for 1 h followed by 10 μM CTN for a further 24 h. The developmental status of blastocysts was observed in culture on fibronectin-coated dishes for 72 h during post-treatment and used to identify blastocysts attached to fibronectin-coated dishes only (attachment only), classified as ICM+, ICM++, and ICM+++. Morphological assessment was based on the criteria described in Materials and Methods. The total blastocyst number was 150 for each group. Values are presented as means ± SD of five determinations. Different symbols indicate significant differences at *p* < 0.05.

The in vitro effects of LQ and CTN on embryonic development were further investigated. Our data showed that ~85% of cultured morulae developed into blastocyst-stage embryos in vitro (Figure 2A). The development potential of blastocysts from morulae was reduced in the 10 μM CTN-treated group. However, pretreatment with 20–40 μM LQ significantly prevented this impairment (Figure 2A). The protective effect of LQ on the in vitro development potential of blastocysts treated with 10 μM CTN was further analyzed. In terms of the outgrowth status of blastocysts in vitro, most blastocysts in the control group attached and expanded on fibronectin-coated dishes (developmental status, ICM+++) whereas fewer blastocysts displayed this behavior after treatment with 10 μM CTN (most blastocysts either attached on fibronectin-coated dishes only or further developed to the ICM+ stage) (Figure 2B). This CTN-induced impairment of developmental potential (attachment and outgrowth) was effectively prevented by LQ pretreatment (Figure 2B).

### 2.3. Effect of LQ on the Disruption of Blastocyst Development by CTN in the Embryo Transfer Assay

**Figure 3:**
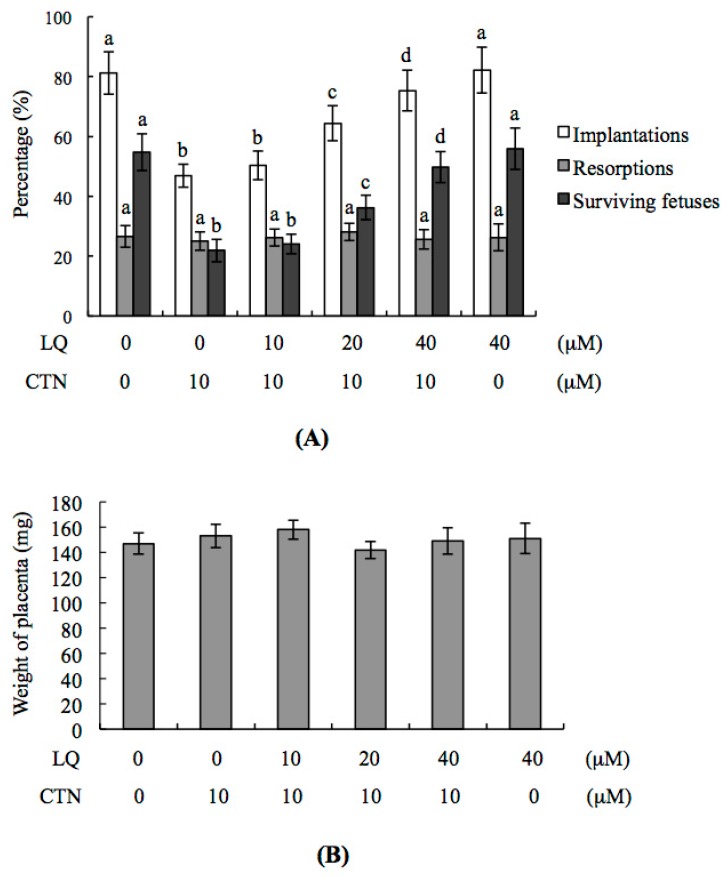

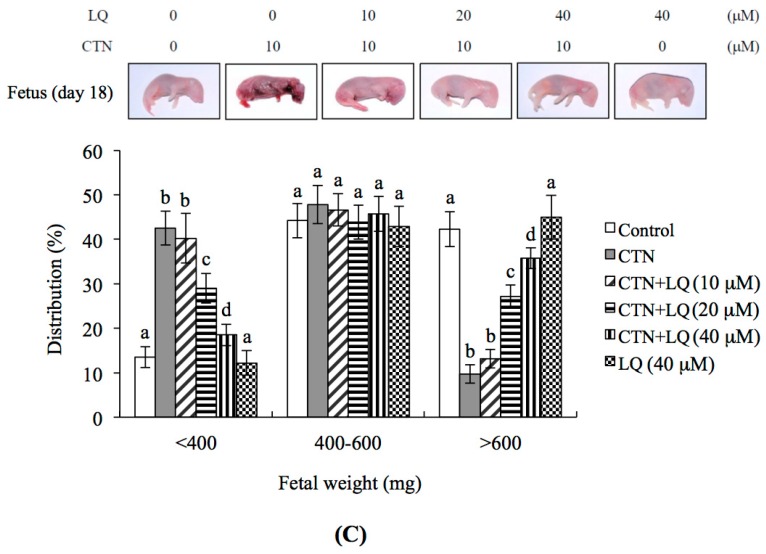
Effects of LQ on implantation, resorption, fetal survival and fetal weight in CTN-treated blastocysts via embryo transfer assay. (**A**) Mouse blastocysts were preincubated with LQ (10–40 μM). After 1 h, embryos were further treated with or without 10 μM CTN for another 24 h. Medium containing 0.5% DMSO was used as the control (vehicle group). Embryos were further transferred to pseudopregnant recipient mice. The percentages of implantations, resorptions and surviving fetuses represent the number of implantations, resorptions and surviving fetuses per number of transferred embryos × 100, respectively; (**B**) Placental weights of 40 pseudopregnant recipient mice were examined; (**C**) The weight distribution of each group of surviving fetuses on day 13 post-transfer (day 18 post-coitus) was measured as described in Materials and Methods (320 total blastocyst transfer to 40 recipients). Different symbols indicate significant differences at *p* < 0.05.

To further investigate the effects of LQ and CTN on blastocyst development in vivo, we transferred control or mouse blastocysts pretreated with 10 μM CTN, and examined the uterine content during post-implantation development at day 18 post-coitus (13 days post-transfer). The implantation ratio of CTN-treated embryos was lower than that in the vehicle group and this impairment was significantly prevented by pretreatment with LQ (Figure 3A). Moreover, implanted embryos failed to develop normally, resulting in embryo resorption in utero in CTN-treated embryos, but not to a significant extent compared to the untreated control group (Figure 3A). The ratios of surviving fetuses of transferred embryos were significantly lower in the CTN-treated than control group (Figure 3A). Pretreatment with 20–40 μM LQ markedly attenuated CTN-induced hazardous effects on embryonic development in the embryo transfer assay model (Figure 3A). Interestingly, no significant differences in placental weight were observed between 10 μM CTN-treated and control groups (Figure 3B). We assessed fetal morphology at day 18 of the embryo transfer assay. Notably, no significant differences in the morphology of fetuses were observed among CTN+LQ-treated, LQ-treated and control groups. However, the average fetal weight of 10 μM CTN-treated groups was lower than that of the untreated control group (Figure 3C). Earlier studies suggest that fetal weight is an important indicator of developmental status [20,29,30,31,32]. Our findings clearly highlight the potential of CTN to cause post-implantation development injury. Furthermore, the percentage of fetal weights <400 mg in the 10 μM CTN-treated group was higher than that in the control group (Figure 3C). Taken together, the results confirm that the CTN-treated group had poorer fetal development (<400 mg) than the control group. Notably, the percentage of fetuses weighing >600 mg was significantly lower in the 10 μM CTN-treated than the untreated control group (Figure 3C). However, CTN-induced impairment of the post-implantation development status (based on average fetus weight) was rescued by pre-treatment with 20 and 40 μM LQ (Figure 3C).

### 2.4. Mechanism of LQ Activity against MG-Induced Development Injury in Mouse Blastocysts

**Figure 4:**
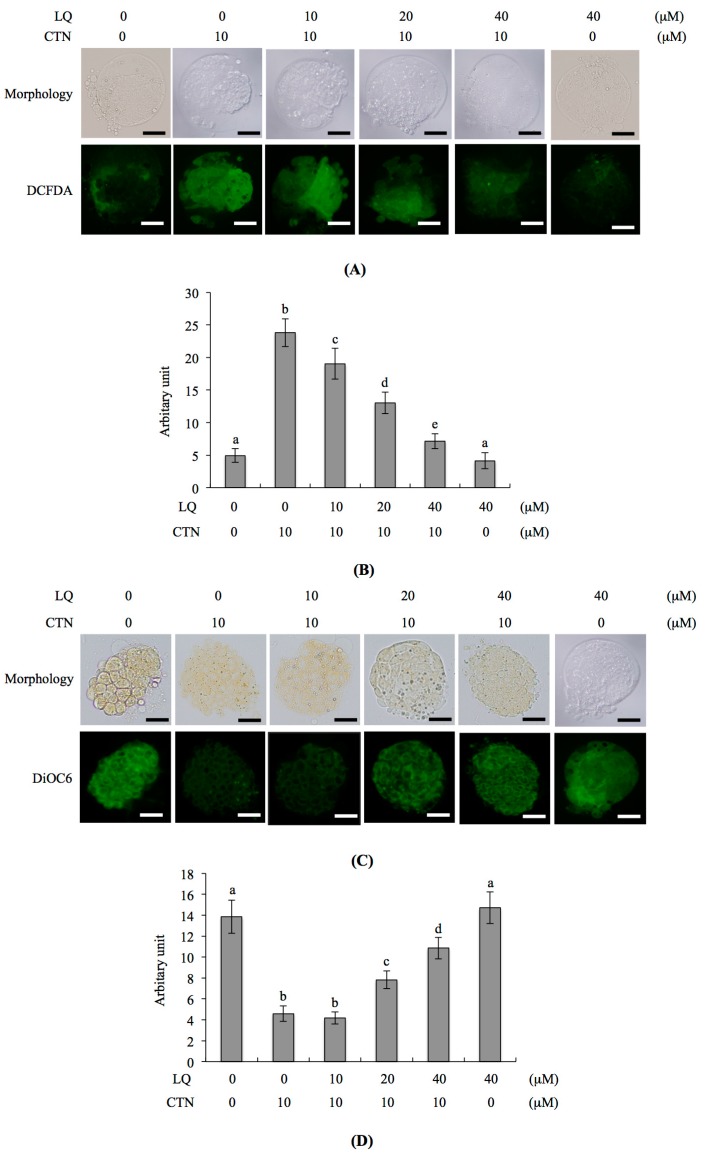
Effects of LQ on CTN-induced reactive oxygen species (ROS) generation and loss of mitochondrial membrane potential in mouse blastocysts. (**A**) Mouse blastocysts were preincubated with LQ (10–40 μM). After 1 h, embryos were further treated with or without 10 μM CTN for a further 24 h. (**A**) DCF-DA (2′,7′-dichlorodihydrofluorescein diacetate) fluorescence dye (20 μM) was used to detect intracellular ROS generation; (**B**) Quantitative analysis of intracellular ROS production in each group was conducted using image J software; (**C**) Embryos were incubated with 40 nM DiOC6(3) at 37 °C for 1 h and mitochondrial membrane potential (MMP) changes examined. MMP was analyzed under a fluorescence microscope; (**D**) Quantitative analysis of MMP changes in each group was conducted using image J software. The total blastocyst number was 160 for each group. Values are presented as means ± SD of five determinations. Different symbols indicate significant differences at *p* < 0.05. The scale bar is 20 μm.

**Figure 5:**
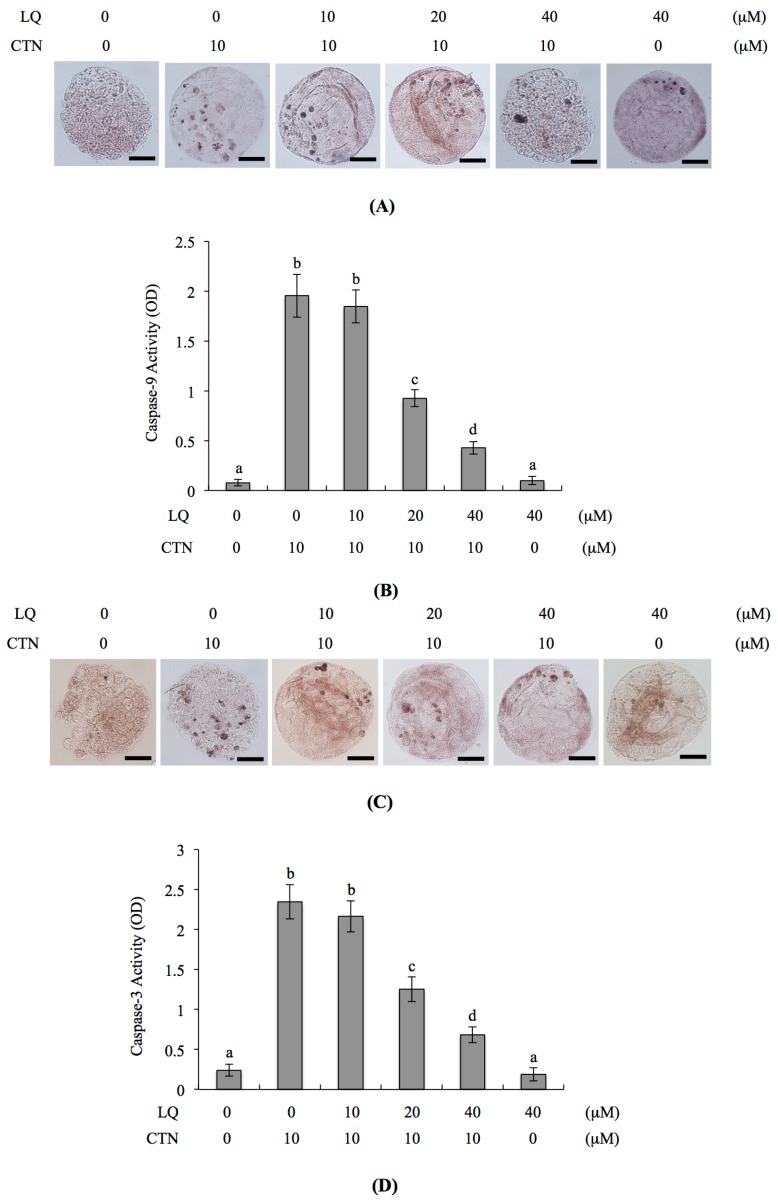
Effects of LQ on caspase-9 and caspase-3 activities in CTN-triggered mouse blastocysts. Mouse blastocysts were preincubated with LQ (10–40 μM) for 1 h. Embryos were further treated with or without 10 μM CTN for 24 h. (**A**,**C**) Activation of caspase-9 and caspase-3 was analyzed by immunostaining with caspase-9 and caspase-3 antibodies for 3 h, followed by secondary antibodies conjugated to peroxidase for 1 h. Diaminobenzidine (DAB) solution was added to embryos as the peroxidase substrate for color rendering. Activated caspase-9 and caspase-3 are presented in black; (**B**,**D**) Activities of caspase-9 (**B**) and -3 (**D**) of blastocysts were measured using the caspase assay kit. Data are expressed as arbitrary units. Values are presented as means ± SD of five determinations. Different symbols indicate significant differences at *p* < 0.05. The scale bar is 20 μm.

We further investigated the mechanisms underlying the beneficial effects of LQ on CTN-triggered apoptosis and development injury. Several researchers to date have demonstrated that ROS induces apoptosis [7,26,33,34]. Moreover, CTN triggers apoptosis through ROS generation [28]. In the current study, dichlorodihydrofluorescein diacetate (DCHF-DA) fluorescent dye was used to measure the intracellular ROS content in LQ- and CTN-treated mouse blastocysts. The fluorescence intensity was increased in the CTN-treated group, compared with untreated control cells, and pretreatment with 20 and 40 μM LQ effectively prevented CTN-caused ROS generation (Figure 4A,B). Further exploration of the effects of LQ on mitochondrial membrane potential (MMP) in CTN-treated mouse blastocyst cells revealed that pre-treatment with 20–40 μM LQ effectively blocked the reduction of DiOC_6_(3) uptake into mitochondria, an MMP assay parameter, clearly highlighting significant protection against MMP loss in blastocysts (Figure 4C,D). Moreover, activation of caspase-9 and caspase-3, important indicators of intrinsic apoptotic signaling cascades, following treatment with 10 μM CTN was significantly inhibited upon pre-treatment with 20–40 μM LQ (Figure 5A–D). Based on the results, we suggest that CTN triggers ROS generation for sequent activation of mitochondria-dependent intrinsic apoptotic processes in mouse blastocyst cells. LQ attenuates CTN-triggered apoptosis and injury to blastocysts through scavenging ROS, an upstream apoptotic inducer.

### 2.5. Effect of LQ on CTN-Induced Blastocyst Development Disruption in an Animal Model

**Figure 6:**
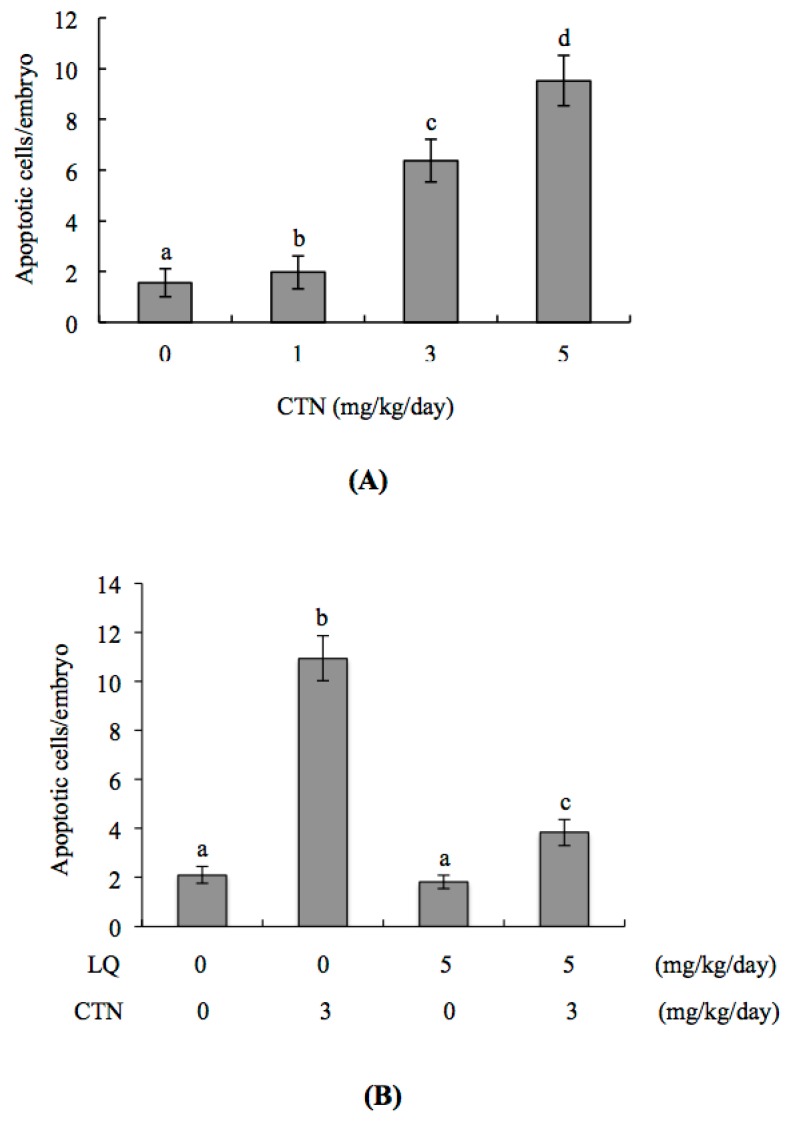

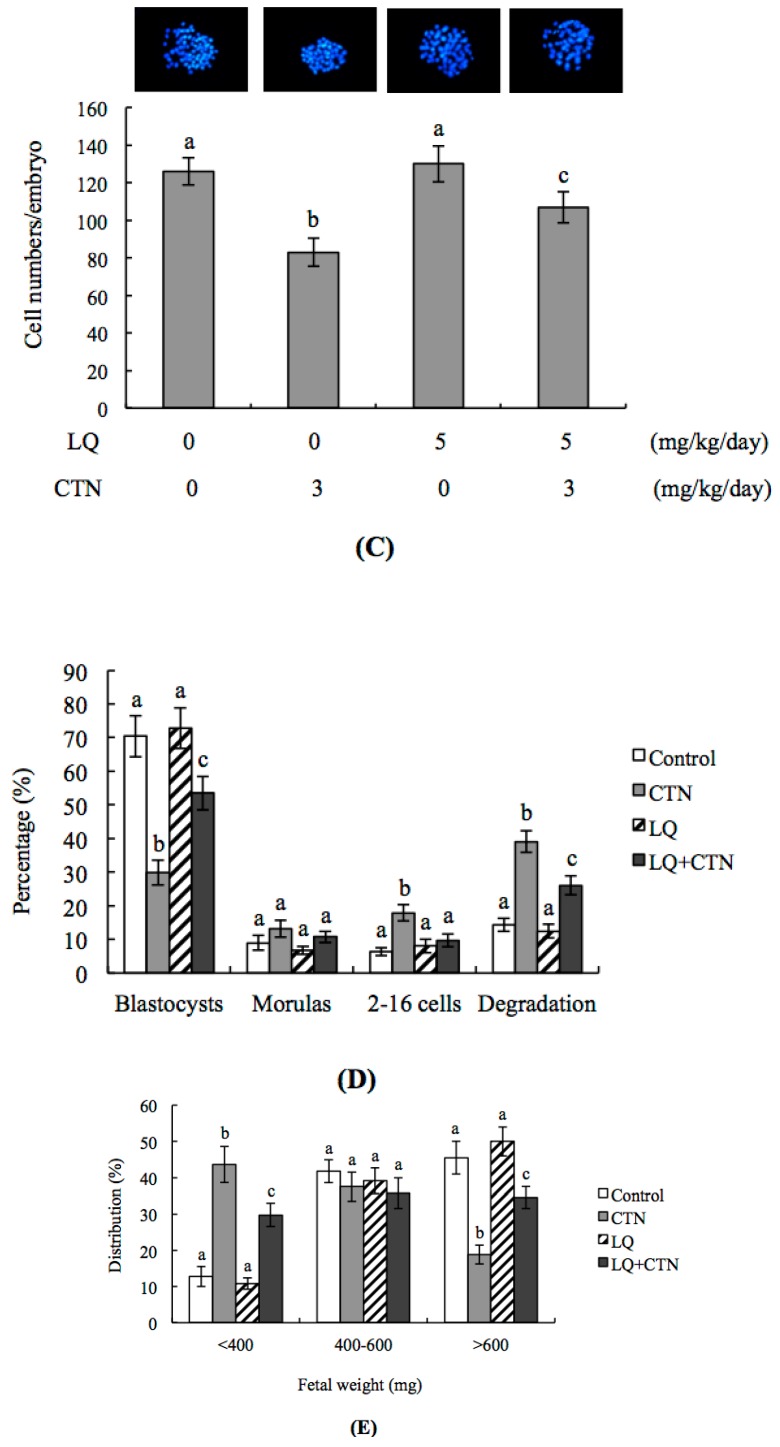
Effects of intravenous injection with LQ and CTN on apoptosis, cell number, pre- and post-implantation embryonic development in an animal model. Twenty randomly selected female mice were intravenously injected with LQ (5 mg/kg/day) for four days and further with LQ (5 mg/kg/day) and CTN (3 mg/kg/day). PBS containing 0.5% DMSO was used as the control (vehicle group). At 24 h after LQ and CTN injection, female mice were mated with male mice overnight. Female mice were continuously intravenously injected with LQ and CTN for an additional four days, and blastocysts obtained by flushing the uterine horn on day four after mating. (**A**,**B**) TUNEL-positive (apoptotic) cells in LQ- and/or CTN-injected (dosage as indicated) blastocysts were detected. The mean number of apoptotic cells per blastocyst was counted; (**C**) Hoechst 33258 staining was used to count the total cell number per blastocyst under a light microscope; (**D**) Developmental stages of embryos obtained from mouse uterine horns on day four were examined based on morphological analyses under a light microscope; (**E**) The weight distribution of each group of surviving fetuses on day 18 post-coitus was measured. Values are presented as means ± SD of five determinations. Symbols indicate significant differences at *p* < 0.05.

Finally, we investigated the effects of LQ on the CTN-triggered disruption of blastocyst development in an animal model by intravenously injecting female mice with LQ (5 mg/kg/day) and CTN (0, 1, 3, and 5 mg/kg/day). Marked apoptosis of blastocysts was evident in female mice injected with 3 and 5 mg/kg/day of CTN (Figure 6A). In addition to apoptosis, we observed lower cell proliferation and the disruption of embryonic development from the zygote to blastocyst stages in the CTN (3 mg/kg/day) injection group (Figure 6B–D). Importantly, pre-injection of LQ (5 mg/kg/day) one day before CTN injection effectively reduced cell apoptosis and impaired the effects of CTN on development (Figure 6B–D). Moreover, assessment of the uterine content of 3 mg/kg/day CTN-injected animals revealed not only development potential impairment from the zygote to blastocyst stage but also fetal weight decrease, compared to that in control (vehicle-injected) mice (Figure 6D,E). LQ injection consistently attenuated the CTN-induced decrease in fetal weight (Figure 6E). Our findings collectively suggest that CTN has the potential to reduce pre-implantation and post-implantation development in vivo, and LQ can effectively rescue CTN-triggered apoptosis and sequent embryonic development injury.

## 3. Discussion

Contamination by fungal toxins, in particular, the mycotoxin CTN, is commonly detected in various foodstuffs and animal feeds. CTN contamination has been reported in *Monascus* fermentation products commonly used as food colorants and flavor enhancers in the Orient [4,5]. Recent studies have further documented the use of *Monascus* fermentation products as dietary supplements to prevent heart disease and decrease plasma triglyceride and cholesterol levels [3]. The reported levels of CTN in cereal grains and fermented maize dough are ~180–580 ng/g [29]. Moreover, lipid extracts of commercialized *Monascus* products are contaminated with 0.28 to 6.29 μg/g CTN [4]. The IC_50_ value of CTN contamination of lipid extracts of *Monascus* products was determined as 60 μM during the course of treatment of human HEK 293 cells for 72 h [4]. In earlier studies, 15–30 μM of CTN induced apoptosis in mouse blastocysts, reducing the cell number and retarding early postimplantation development [6]. Moreover, the incubation of mouse oocytes with 5 μM CTN caused impairment of the maturation, fertilization, and sequential embryonic development in vitro and in vivo [8]. Here, we examined the protective effects of LQ against cytotoxicity mediated by 10 μM CTN (~1.25 μg/g in culture medium), a treatment dosage reflecting its concentration in contaminated foods that induces apoptosis and the disruption of blastocyst development.

Accumulating experimental evidence has highlighted a critical role of LQ as a scavenger of intracellular ROS that inhibits or regulates ROS-mediated signaling pathways to protect various cell types against injury or damage, including human umbilical vein endothelial cells (HUVECs), neurons, hippocampal neurons, macrophages, hepatocytes and osteoblasts [19,21,30,31,32]. The intracellular ROS level is a key regulator of apoptotic processes in several cell models [35,36]. Under normal physiological conditions, intracellular ROS are involved in the determination of cell fate via the activation of transcription factors and a series of signaling cascades that control proliferation and differentiation [37]. However, apoptosis and damage may be triggered when cells produce ROS that exceeds the capacity of the cellular redox buffering system to lower elevated ROS to the normal basal level. Increased intracellular ROS levels lead to massive Ca^2+^ influx, disruption of lipoxygenase (LOX) activity, lipid peroxidation, mitochondrial dysfunction and cell death (apoptosis or necrosis). In addition, superoxide anion (O^2−^), the precursor of H_2_O_2_, OH^−^ and other ROS, may trigger cellular biomolecule damage via oxidation reactions with proteins, DNA and lipids, causing harmful effects, and in turn, activating cell death signaling processes [37]. Several studies have demonstrated that LQ can either stimulate or inhibit apoptotic signaling [14,15,16,19,38]. Data from the current study indicate that LQ blocks CTN-induced ROS generation and various apoptotic parameters in mouse blastocysts at doses greater than 20 μM (Figure 4 and Figure 5). Specifically, our experiments showed for the first time that LQ effectively attenuates CTN-induced injurious effects on embryonic development through the blockage of excess intracellular ROS formation (Figure 3, Figure 4, Figure 5 and Figure 6). Thus, the ambiguous effects of LQ could depend on the specificity of the target cell type, treatment period and dosage. However, the precise regulatory mechanisms of action of LQ on cellular apoptosis require further investigation.

Embryonic development is a complex and precisely-regulated process. Various environmental teratogens (chemical, physical or biological factors) can trigger embryonic developmental injury and the development of effective protective strategies is therefore an important issue in the field of reproductive toxicology. Previous studies by our group have demonstrated that CTN induces apoptosis, causing harmful effects on oocyte maturation and fertilization, impairs blastocyst development from morula, and promotes the early-stage cell death of mouse blastocysts [6,7,8,33]. Blastocysts are composed of two different cell types, trophectoderm (TE) and ICM. TE cells essentially develop as a sphere of epithelial cells surrounding the ICM and blastocoel and contribute to placenta formation for further fetal development of the mammalian conceptus [39]. Reduction of the TE cell number is reported to exert detrimental effects on embryonic implantation and sequent development [6,40,41,42,43,44]. ICM cells have the potential to develop into the fetus and a reduction in the number of these cells may thus have a deleterious impact on post-implantation embryonic development and fetus formation. ICM and TE cells show different sensitivities to apoptotic inducers examined in previous studies by our group [45,46,47,48]. In the current investigation, only the ICM component displayed cell number reductions and apoptotic processes in 10 μM CTN-treated mouse blastocysts. To our knowledge, this study has shown for the first time that LQ significantly blocks CTN-induced apoptosis and reduces cell proliferation in mouse blastocysts (Figure 1).

Two major apoptotic signaling pathways have been identified, specifically, intrinsic and extrinsic pathways. The extrinsic apoptotic pathway is triggered via extracellular-death-inducing, ligand-specific binding with membrane death receptors that stimulates caspase-8 activation, followed by the activation of downstream apoptotic cascades. The intrinsic apoptotic pathway is activated by DNA damage or the generation of free radicals, triggering a mitochondria-dependent apoptotic cascade involving caspase-9 activation and downstream apoptotic processes. Mitochondria-dependent apoptotic regulatory mechanisms are further classified into caspase-dependent and -independent pathways. In the caspase-dependent pathway, MMP loss induces cytochrome c release from the mitochondria to cytosol, and caspase-9 is subsequently activated to trigger the downstream caspase-dependent signaling cascade. In the caspase-independent signaling pathway, apoptosis-inducing factor (AIF) is critically involved [49]. Here, we demonstrated that CTN-mediated apoptosis of blastocysts occurs through intrinsic mitochondria- and caspase-dependent pathways involving ROS generation, MMP loss, and the activation of caspase-9 and caspase-3 (Figure 4 and Figure 5). LQ effectively prevented CTN-induced ROS generation, MMP loss and caspase-9 and caspase-3 activation (Figure 4 and Figure 5). The ability of LQ to protect against CTN-induced developmental injury was further confirmed in an animal model in vivo (Figure 6).

## 4. Materials and Methods

### 4.1. Chemicals and Reagents

Pregnant mare serum gonadotropin (PMSG), bovine serum albumin (BSA), sodium pyruvate, citrinin and liquiritigenin were purchased from Sigma (St. Louis, MO, USA). Human chorionic gonadotropin (hCG) was obtained from Serono (NV Organon, Oss, The Netherlands). The TUNEL in situ cell death detection kit was purchased from Roche (Mannheim, Germany) and CMRL-1066 medium from Gibco Life Technologies (Grand Island, NY, USA).

### 4.2. Collection of Mouse Morulae and Blastocysts

Institute of Cancer Research (ICR) mice were purchased from Taiwan National Laboratory Animal Center (Taipei, Taiwan). All experiments were approved by the Animal Research Ethics Board of Chung Yuan Christian University (Taiwan). Humane care was provided for the animals following the guidelines for the Guide to the Care and Use of Experimental Animals (Canadian Council on Animal Care, Ottawa, 1993; ISBN: 0-919087-18-3). All ICR mice were maintained on breeder chow (Harlan Teklad) with food and water available *ad libitum* and housed in standard 28 cm × 16 cm × 11 cm (height) polypropylene cages with wire-grid tops under a 12 h night/12 h day regimen. Superovulation of mice was performed through intraperitoneal injection with 5 international unit (IU) PMSG. After 48 h, mice were further subjected to intraperitoneal injection with 5 IU hCG, followed by mating overnight with a single fertile male ICR mouse. The morning a vaginal plug was identified was defined as day zero of gestation. Plug-positive female mice were used for further experimentation. On the afternoon of gestation day three, mice were sacrificed and the morulae collected by flushing the uterine tubes. Blastocysts were obtained by flushing the uterine horn on day four. The flushing solution for the morulae or blastocysts consisted of CMRL-1066 medium containing 1 mM glutamine and 1 mM sodium pyruvate. The morulae and blastocysts collected from different females were pooled and randomly divided into several groups for further experiments.

### 4.3. Analysis of Blastocysts Developing from Morulae

Morulae were pre-incubated in medium containing 10–40 μM LQ for 1 h and treated with CTN (2.5, 5 or 10 μM) for a further 24 h at 37 °C for embryo development. CTN treatment performed with a final DMSO concentration of up to 0.5% (*v*/*v*). Medium containing 0.5% DMSO was used as the control (vehicle group). The number of blastocyst-stage embryos derived from the morulae was counted and the percentages of morulae developing into blastocysts estimated using phase-contrast microscopy (Olympus BX51, Tokyo, Japan).

### 4.4. TUNEL Assay of Blastocysts

CTN and LQ stock solutions were dissolved in dimethyl sulfoxide (DMSO) and stored at −30 °C. To determine the effects of LQ and CTN on embryos, blastocysts were pre-incubated in medium containing 10–40 μM LQ for 1 h, and CTN treatment performed (2.5, 5 or 10 μM) for a further 24 h with a final DMSO concentration of up to 0.5% (*v*/*v*). Medium containing 0.5% DMSO was used as the control (vehicle group). For the detection of DNA fragmentation, embryos were washed with LQ and CTN-free medium and fixed in 4% paraformaldehyde (PFA) at room temperature. After 2 h, embryos were permeabilized and subjected to terminal deoxynucleotidyl transferase dUTP nick end labeling (TUNEL) using an in situ cell death detection kit (Roche Molecular Biochemicals, Mannheim, Germany). All experimental procedures were performed according to the manufacturers’ protocols. In brief, each group of blastocysts was treated with 20 μL TUNEL reaction mixture (2 µL enzyme solution mix with 18 µL labeling solution containing fluorescein-conjugated nucleotides) for 30 min at 37 °C. To remove the TUNEL reaction mixture, embryos were washed three times with phosphate-buffered saline (PBS) containing 0.3% (*w*/*v*) BSA. Each group of embryos was incubated with converted 20 µL peroxidase (POD) solution for 30 min at 37 °C. Embryos were extensively re-washed with PBS to remove the POD solution and further incubated with 20 µL DAB (3,3′-diaminobenzidine) substrate solution for 3 min at room temperature. TUNEL-positive cell images (black spots) were obtained via fluorescence microscopy under bright light.

### 4.5. Measurement of Embryo Cell Numbers

Dual differential staining was employed to facilitate the counting of cell numbers of blastocysts in the inner cell mass (ICM) and trophectoderm (TE) according to a previously published protocol [44]. In brief, blastocysts were incubated in 0.4% pronase in M_2_–BSA medium (M_2_ containing 0.1% bovine serum albumin) to remove the zona pellucida. Denuded blastocysts were further exposed to 1 mM trinitrobenzenesulphonic acid (TNBS) in BSA-free M_2_ medium containing 0.1% polyvinylpyrrolidone (PVP) at 4 °C for 30 min and washed with M_2_, in keeping with previously published procedures [50]. Next, blastocysts were incubated with 30 μg/mL anti-dinitrophenol-BSA complex antibody in M_2_-BSA at 37 °C for 30 min. Embryos were co-incubated with M_2_ supplemented with 10% whole guinea pig serum containing complement along with 20 μg/mL bisbenzimide and 10 μg/mL propidium iodide (PI) at 37 °C for 30 min. Immunolysed blastocysts were gently transferred to slides and protected from light before observation under a fluorescence microscope. Under UV light excitation, ICM cells (which take up bisbenzimidine but exclude PI) appeared blue whereas TE cells (which take up both fluorochromes) appeared orange-red. The number of nuclei counted was taken as representing the number of cells because during preimplantation stage mouse embryos are not usually multinucleate [51].

### 4.6. In Vitro Embryonic Development Analysis

The in vitro embryonic development was assessed according to a modified method reported previously [52]. In brief, each well of fibronectin-coated, four-well multidishes was cultured with four blastocyst-stage embryos at 37 °C. The in vitro culture medium for embryonic development (hereafter designated “culture medium”) was CMRL-1066 containing 1 mM glutamine and 1 mM sodium pyruvate plus 50 IU/mL penicillin and 50 mg/mL streptomycin. Embryos were cultured for three days in medium supplemented with 20% fetal calf serum and four days in medium supplemented with 20% heat-inactivated human placental cord serum. The embryo development status was assessed and images obtained daily under a phase-contrast dissecting microscope. Embryo developmental stages were classified according to previously established criteria [53,54]. Under these culture conditions, each hatched blastocyst attached to fibronectin and grew to form a cluster of ICM cells over the trophoblastic layer via a process known as TE outgrowth. Embryo outgrowth status was measured during a total in vitro culture period of 72 h to estimate morphological scores. Embryo development status was classified as either “attached” or “outgrowth”. Embryonic outgrowth was defined by the presence of a cluster of ICM cells over the trophoblastic layer. The morphological scores were estimated according to ICM clusters based on shape, ranging from compact and rounded (+++) to a few scattered cells (+) over the trophoblastic layer [55,56].

### 4.7. Blastocyst Development Following Embryo Transfer

Blastocyst development was evaluated with a nonsurgical embryo transfer (NSET) device for the transfer of blastocysts using a previously reported method [57]. To investigate the ability of expanded blastocysts to implant and develop in vivo by embryo transfer, each group of generated embryos was transferred to 40 pseudopregnant recipient mice. Pseudopregnant dams were generated by the mating of white skin color ICR female mice with vasectomized black skin color males (C57BL/6J). One vasectomized fertile male mouse was used to mate one ICR female and a total of 40 pseudopregnant dams were produced as recipients for embryo transfer. The skin color of fetuses was assessed at day 18 post-coitus to ensure that all fetuses were obtained from embryo transfer (white color) and not fertilization by C57BL/6J (black color). To examine the impacts of LQ and CTN on postimplantation growth in vivo, eight LQ- and/or CTN-treated blastocysts were transferred to the right uterine horn and untreated control (vehicle) group embryos were transferred in parallel to the left uterine horn of pseudopregnant mice on day four. Surrogate mice were sacrificed on day 18 post-coitus (day 13 post-transfer). Embryo implantation rates were calculated as the number of implantation sites per number of embryos transferred. The resorption and survival rates were calculated as the number of resorbed or surviving fetuses, respectively, per number of fetuses transferred. The weights of the placenta and surviving fetuses were measured immediately after sacrifice.

### 4.8. Intravenous Injection of Female Mice with LQ and CTN

To examine the effects of LQ and CTN on mouse embryonic development in an animal model, 20 randomly selected female (42 days old) mice were exposed to various doses of LQ via intravenous tail injection for four days and further treated with LQ or CTN as indicated. After 24 h, injected ICR female mice were mated overnight with a single fertile ICR male. Mice were intravenously injected with the indicated doses of LQ and/or CTN continuously for four days after mating and the blastocysts collected by flushing the uterine horn on day four. The cell proliferation and apoptosis of blastocysts, as well as the embryonic development and fetus weights were examined.

### 4.9. Measurement of ROS in Mouse Blastocysts

ROS in embryos was detected according to a previously documented protocol [43]. In brief, mouse blastocysts were pre-treated with 20 μM DCF-DA for 1 h followed by incubation with LQ and/or CTN for 24 h. ROS generation was examined under an Olympus BX51 fluorescence microscope. Intracellular ROS generation in each group was quantitatively analyzed using Image J software.

### 4.10. Detection of Mitochondrial Membrane Potential (MMP) in Mouse Blastocysts

The MMP of embryos was detected using a previously published protocol [43]. In brief, mouse blastocysts were treated with or without LQ and/or CTN for 24 h and subsequently incubated with DiOC6(3) fluorescence dye (50 nM) for 15 min. Fluorescence was detected under the Olympus BX51 fluorescence microscope. MMP was quantified using Image J software.

### 4.11. Measurement of Caspase Activity

The caspase activity of embryos was detected as previously described [43]. In brief, the activity of caspase-9 and -3 in embryos was measured using the caspase assay kit (R&D Systems, Minneapolis, MN, USA). Cell lysates of blastocysts were added to the reaction mixtures containing dithiothreitol and caspase substrates (for -9 and -3), and incubated for 1 h at 37 °C according to the manufacturer’s instructions. A fluorescence ELISA (enzyme-linked immunosorbent assay) reader was used to measure the absorbance of the developed chromophore with excitation at 405 nm.

### 4.12. Statistical Analysis

Data were analyzed using one-way analysis of variance (ANOVA) followed by Dunnett’s test for multiple comparisons, and presented as means ± SD (standard deviation). Different symbols represent significant differences at *p* < 0.05.

## 5. Conclusions

In conclusion, LQ, a natural polyphenolic flavonoid compound, prevents CTN-caused embryonic development injury via the blockage of ROS generation, supporting the potential utility of this flavonoid compound for development as a health food supplement to prevent CTN-induced teratogenic effects.
